# Depression 12-months after coronary artery bypass graft is predicted by cortisol slope over the day

**DOI:** 10.1016/j.psyneuen.2016.05.025

**Published:** 2016-09

**Authors:** Lydia Poole, Tara Kidd, Amy Ronaldson, Elizabeth Leigh, Marjan Jahangiri, Andrew Steptoe

**Affiliations:** a Department of Epidemiology and Public Health, University College London, 1-19 Torrington Place, London WC1E 6BT, United Kingdom; b Department of Cardiac Surgery, St George’s Hospital, University of London, Blackshaw Road, London SW17 0QT, United Kingdom

**Keywords:** Depression, Cortisol, Coronary artery bypass graft surgery, Longitudinal

## Abstract

Alterations in the diurnal profile of cortisol have been associated with depressed mood in patients with coronary heart disease. The relationship between cortisol output and depressed mood has not been investigated prospectively in coronary artery bypass graft (CABG) patients before. We aimed to study the relationship between cortisol measured pre- and post-operatively and depression symptoms measured 12 months after CABG surgery. We analysed data from 171 patients awaiting first-time, elective CABG surgery from the pre-assessment clinic at St. George’s Hospital, London. The Beck Depression Inventory (BDI) was used to assess depression symptoms and saliva samples were collected to measure diurnal cortisol. Baseline assessments of depression and cortisol were obtained an average 29 days before surgery, short-term follow-up of cortisol occurred 60 days after surgery and long-term follow-up of depression was assessed 378 days after surgery. Baseline cortisol slope was not associated with depression at 12-month follow-up. However, a steeper cortisol slope measured 60 days after surgery predicted reduced odds of depression (BDI ≥ 10) 12 months after surgery (odds ratio 0.661, 95% confidence interval 0.437–0.998, *p* = 0.049) after controlling for covariates. These findings suggest interventions aimed at improving adaptation in the early recovery period may have long-term benefits in this patient group.

## Highlights

• We present data from 171 elective coronary artery bypass graft patients.
• We measured diurnal cortisol one month before and two months after surgery.
• Cortisol slope two months after surgery predicted depression 12 months later.
• These results suggest short-term surgical recovery is important for later depression.

## 1 Introduction

Cortisol is implicated in the pathophysiology of depression (Zunszain et al., 2011). Hypothalamic-Pituitary-Adrenal (HPA) axis abnormalities have been observed in patients with major depression, including increased secretion and reactivity of cortisol (Cowen, 2010), elevated corticotropin releasing hormone (Nemeroff et al., 1984), and increased size and activity of the pituitary and adrenal glands (Nemeroff et al., 1992). Elevated cortisol has also been linked to cardiac risk factors (Brown et al., 2004) and we recently reported that dysregulated diurnal cortisol profiles prior to coronary artery bypass graft (CABG) surgery predicted increased risk of death or adverse cardiac events in subsequent years (Ronaldson et al., 2015).

Alterations in the diurnal profile of cortisol have been associated with depressed mood in patients with coronary heart disease (Bhattacharyya et al., 2008), and with history of depression in acute coronary syndrome patients (Messerli-Bürgy et al., 2012). HPA axis dysregulation has also been associated with greater cardiac-related mortality in depressed patients (Jokinen and Nordström, 2009). There are limited studies investigating cortisol in CABG patients, although there is some evidence of heightened cortisol output in the post-operative period (Gibbison et al., 2015; Roth-Isigkeit and Schmucker, 1997). The relationship between cortisol output and depressed mood has not been investigated in CABG patients before. We aimed to study the prospective relationship between cortisol rhythm across the day measured pre- and post-operatively and depression symptoms measured 12-months after CABG surgery.

## 2 Method

### 2.1 Sampling and procedure

Full details of the methods and participants used in the ARCS (Adjustment and Recovery after Cardiac Surgery) study can be found elsewhere (Poole et al., 2014, Ronaldson et al., 2015). Briefly, we conducted complete case analysis on data from 171 patients awaiting first-time, elective CABG surgery, who were recruited from the pre-assessment clinic at St. George’s Hospital, London. Inclusion criteria permitted only patients who were undergoing elective CABG surgery or CABG plus valve replacement to participate. CABG surgery was defined to include both on-pump (i.e. cardiopulmonary bypass) and off-pump surgical procedures. In addition, participants had to be able to complete the questionnaires in English, and be 18 years or older. The ARCS study was powered to assess the importance of a broad range pf psychological, behavioural, and social factors. On average, baseline assessments occurred 29 days before surgery, short-term follow-up occurred 60 days after surgery and long-term follow-up was 378 days after surgery. All procedures were carried out with the written consent of the participants. We obtained ethical approval from the South West London research ethics committee.

### 2.2 Measures

#### 2.2.1 Predictor variable: cortisol slope

Full details of the saliva collection protocol has been described elsewhere (Ronaldson et al., 2015). In brief, the saliva samples were collected using Salivettes (Sarstedt, Leicester, UK) at set time points across the day: on awakening, 30 min after awakening, 10am, midday, 4pm, 8pm and bedtime. Saliva samples were obtained at baseline and short-term follow-up; they were returned via post and stored at −20 ° Celsius for analysis at a later date. Cortisol levels were assessed using a time resolved immunoassay with fluorescence detection, at the University of Dresden. The intra- and inter-assay coefficients of variation were less than 4%. The slope of cortisol decline over the day was calculated as the reduction in cortisol per hour (nmol/L/hour), using regression methods. The slope excluded the 30-min post-awakening sample (Messerli-Bürgy et al., 2012). Slope values closer to zero reflect flatter diurnal rhythms. Two participants had missing baseline slope values (n = 169).

#### 2.2.2 Outcome variable: depression

The Beck Depression Inventory (BDI) (Beck et al., 1988) was used to measure depression symptoms at baseline and 12 months after surgery. It is a 21-item questionnaire which asks the respondent to reflect on how they have been feeling over the past two weeks. Ratings were summed and we used a standard cut-off of </≥10 to indicate no depression and mild to severe depression respectively.

#### 2.2.3 Covariates

Clinical risk was assessed using the European System for Cardiac Operative Risk Evaluation I (EuroSCORE) (Roques et al., 2003). EuroSCORE is a composite measure of procedural mortality risk. Items were scored in accordance with the ‘logistic EuroSCORE’ method to generate a percentage mortality risk estimate (http://www.euroscore.org/logisticEuroSCORE.htm). Smoking was measured as a binary variable (current smoker/non-smoker). Body mass index (BMI) was assessed at the pre-operative clinic appointment and calculated using the standard formula (kg/m^2^).

### 2.3 Statistical analysis

Associations between variables were assessed using Pearson’s correlations and dependent and independent *t*-tests as appropriate. Cortisol slope data were normally distributed, so the raw data are reported here. Outliers greater than 3 standard deviations from the mean were excluded from the analysis. We used logistic regression analyses to assess the association between cortisol slope and depression status. We controlled for several potential confounders of the associations, namely BMI, smoking status, baseline depression status and EuroSCORE. Age and sex are included in EuroSCORE so were not entered separately to avoid double adjustment. Inclusion of on pump surgery as a covariate did not change the results so is not included in the models presented here. Results are presented as adjusted odds ratios (OR) with 95% confidence intervals (CI). Secondary analyses were performed using continuous BDI scores. All analyses were conducted using SPSS version 21.

## 3 Results

**Fig. 1:**
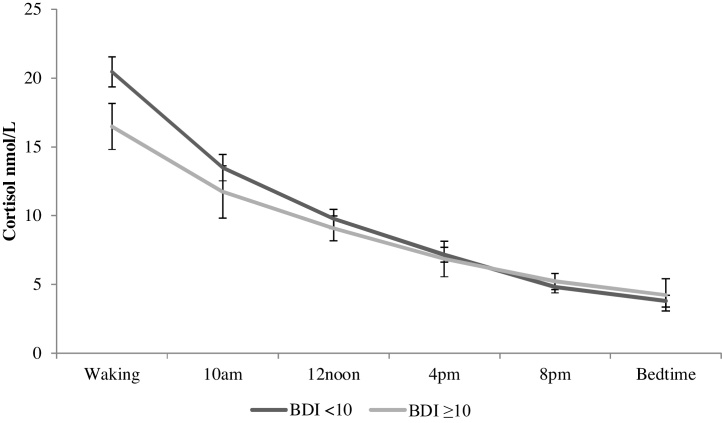
Diurnal cortisol output two months following CABG surgery by depression status at 12-month follow-up. Bars indicate standard errors of the mean.

The demographic and clinical characteristics of the sample are described in Table 1. The majority of ARCS participants were White (90.1%), overweight (BMI > 25 = 79.5%) and male (84.8%). The mean age of the sample was 69.29, with a range of 44–90 years. The mean BDI value at baseline was 8.15 (SD: 6.00) and at 12-month follow-up was 6.33 (SD: 5.69) reflecting an overall decline in depression over time (*t* = 4.296, *p* <0.001). Cortisol slope was slightly higher/steeper at baseline (mean: 1.82 nmol/L/hour, SD: 1.12) than at short-term follow-up (i.e. two months post-CABG) (mean: 1.71 nmol/L/hour, SD: 1.11), but this change was non-significant (*t* = 0.934, *p* = 0.352). Univariate analyses revealed no difference in 12-month depression status by baseline cortisol slope values (*t* = 1.459, *p* = 0.146), so fully adjusted models were not performed. Logistic regression analyses, controlling for covariates revealed short-term follow-up cortisol slope was a significant predictor of depression status 12 months after CABG (OR: 0.661, 95% CI 0.437–0.998, *p* = 0.049). This finding suggests that a one unit increase in slope (i.e. a steeper slope) is associated with a 34% reduction in the odds of depression 12 months after surgery. Baseline depression was also a significant predictor in this model (OR: 8.050, 95% CI 3.525–18.383, *p* < 0.001). This pattern of results was confirmed in multiple linear regression models with short-term follow-up cortisol slope (β = −0.126, *p* = 0.049) and baseline BDI (β = 0.537, *p* < 0.001) predicting continuous BDI scores 12 months after surgery. The relationship between short-term follow-up cortisol output across the day and depression 12 months after surgery is illustrated in Fig. 1.

## 4 Discussion

We found that cortisol slope measured two months after CABG surgery was predictive of depression status 12 months later. These results remained true even after controlling for potential confounders of this relationship, namely BMI, smoking and disease severity. These are the first findings to our knowledge that has implicated cortisol in the manifestation of depression symptoms following CABG surgery.

Previous studies have reported that there is heightened cortisol output in the days following CABG surgery (Gibbison et al., 2015). There are likely multiple factors implicated in the dysregulation of cortisol after surgery, including both physiological mechanisms linked to marked increases in inflammation during the procedure (Biglioli et al., 2003) and recovery periods (Poole et al., 2013), as well as psychological factors such as the experience of stress associated with undergoing a major operation and the related pain, discomfort and reduced mobility. We did not see a significant change in cortisol slope over time, though our results did indicate a reduction in slope (i.e. a flatter slope) at short-term follow-up compared to baseline. It is possible that this small difference in cortisol slope partly explains why we did not observe an association with baseline slope and later depression. Future replication in larger studies is needed to confirm this hypothesis.

In a previous study our group recruited patients with suspected coronary artery disease from hospital outpatient’s clinics prior to diagnostic angiography. Findings showed that a flatter cortisol slope was associated with depression in patients who went on to receive a positive diagnosis for coronary artery disease, but not related to depression in those patients who did not receive a coronary artery disease diagnosis (Bhattacharyya et al., 2008). Otte and colleagues (Otte et al., 2004) also found an association between cortisol and depression in the Heart and Soul study, however the 24-h urinary cortisol samples did not allow diurnal patterns to be explored. Combined, these findings suggest that cortisol dysregulation and depression may be related to the underlying disease processes in cardiac patients; the exact mechanisms for these effects are not yet clear though as alluded to, one possibility draws on the inflammatory model of depression in coronary heart disease (Poole et al., 2011). For example, there is evidence to suggest that pre-operative C-reactive protein levels predict depression experienced 6 months after CABG surgery (Yang et al., 2012) and interferon-gamma measured in the days following surgery predicts depression 12-months after surgery (Steptoe et al., 2015). Inflammatory data was not collected in ARCS at the same post-operative time point as cortisol, preventing this hypothesis from being tested. However, our study is one of the few studies to examine the complex interaction between cardiac disease, cortisol and depression using a CABG population. The findings are clinically significant in that interventions to enhance recovery and reduce stress in the immediate post-operative period may have long-term benefit.

Our study has a number of strengths. The prospective nature of our analyses allows the direction of the effect to be explored, with multiple assessments of cortisol and depression. The ARCS study examined patients undergoing CABG at a single hospital and therefore removes the influence of inter-hospital variation in patient care policy. However, there are also some limitations to our study. First is the reliance on questionnaire measures of depression, since these cannot be used to define clinical depression. Secondly, since our sample was predominately of White ethnicity and male, our findings may not readily generalise to other populations. Next, since we only had a small number of depression cases, this limited the number of covariates we could include in our models. Finally, cortisol was measured over a single day; although this is also the case in the majority of studies of cortisol and health outcomes, values on a single day may be affected by situational factors, increasing variability in findings.

## 5 Conclusions

A steeper cortisol slope measured two months following CABG surgery was associated with reduced odds of depression 12 months later. These findings suggest therapeutic benefit may be attained by intervening in the weeks following cardiac surgery.

## Conflict of interest

None.

## Funding

This research was funded by the British Heart Foundation. The funders had no involvement in the study design; in the collection, analysis and interpretation of data; in the writing of the report; and in the decision to submit the article for publication.

## Contributors

Lydia Poole (PhD), Amy Ronaldson (MSc), Tara Kidd (PhD), Elizabeth Leigh (PhD), Marjan Jahangiri (FRCS (CTh)) and Andrew Steptoe (DSc).

## Figures and Tables

**Table 1:** Demographic, clinical and biological characteristics of the sample (N = 171).

*Characteristic*	*Mean* *±* *SD or N(%)*
*Demographics*	
Age (years)	69.29 ± 8.87
Female	26 (15.2)
Ethnicity − White British/White Other	154 (90.1)
BMI (kg/m^2^)	28.16 ± 3.85
Smoker	10 (5.8)
*Co-morbidities*	
Diabetes	36 (21.1)
Hypertension	134 (78.4)
Pulmonary disease	10 (5.8)
Neurological disorder	11 (6.4)
Extracardiac arteriopathy	12 (7.0)
*Clinical factors*	
Logistic EuroSCORE (%)	4.73 ± 3.21
CABG in isolation	45 (26.3)
Number of grafts	2.85 ± 1.13
On-pump	138 (80.7)
*Depression*	
Baseline BDI (≥10)	55 (32.2)
Long-term follow-up BDI (≥10)	39 (22.8)
*Cortisol*	
Baseline slope (nmol/L/hour)^a^	1.82 ± 1.12
Short-term follow-up slope (nmol/L/hour)	1.71 ± 1.11

a N = 169.
